# Carbon and nitrogen optimization in solid-state fermentation for sustainable sophorolipid production using industrial waste

**DOI:** 10.3389/fbioe.2023.1252733

**Published:** 2024-01-04

**Authors:** Estefanía Eras-Muñoz, Teresa Gea, Xavier Font

**Affiliations:** Department of Chemical, Biological and Environmental Engineering, Escola d’Enginyeria, Composting Research Group (GICOM), Universitat Autònoma de Barcelona, Barcelona, Spain

**Keywords:** biosurfactant, sophorolipid, solid-state fermentation, experimental design, residue revalorization, cosmetic industry, bioconversion

## Abstract

The use of alternative feedstocks such as industrial or food waste is being explored for the sustainable production of sophorolipids (SLs). Microbial biosurfactants are mainly produced via submerged fermentation (SmF); however, solid-state fermentation (SSF) seems to be a promising alternative for using solid waste or byproducts that could not be exploited by SmF. Applying the advantages that SSF offers and with the aim of revalorizing industrial organic waste, the impact of carbon and nitrogen sources on the relationship between yeast growth and SL production was analyzed. The laboratory-scale system used winterization oil cake as the solid waste for a hydrophobic carbon source. Pure hydrophilic carbon (glucose) and nitrogen (urea) sources were used in a Box–Behnken statistical design of experiments at different ratios by applying the response surface methodology. Optimal conditions to maximize the production and productivity of diacetylated lactonic C18:1 were a glucose:nitrogen ratio of 181.7:1.43 (w w^−1^ based on the initial dry matter) at a fermentation time of 100 h, reaching 0.54 total gram of diacetylated lactonic C18:1 with a yield of 0.047 g per gram of initial dry mass. Moreover, time course fermentation under optimized conditions increased the SL crude extract and diacetylated lactonic C8:1 production by 22% and 30%, respectively, when compared to reference conditions. After optimization, industrial wastes were used to substitute pure substrates. Different industrial sludges, OFMSW hydrolysate, and sweet candy industry wastewater provided nitrogen, hydrophilic carbon, and micronutrients, respectively, allowing their use as alternative feedstocks. Sweet candy industry wastewater and cosmetic sludge are potential hydrophilic carbon and nitrogen sources, respectively, for sophorolipid production, achieving yields of approximately 70% when compared to the control group.

## Graphical Abstract

**Figure:**
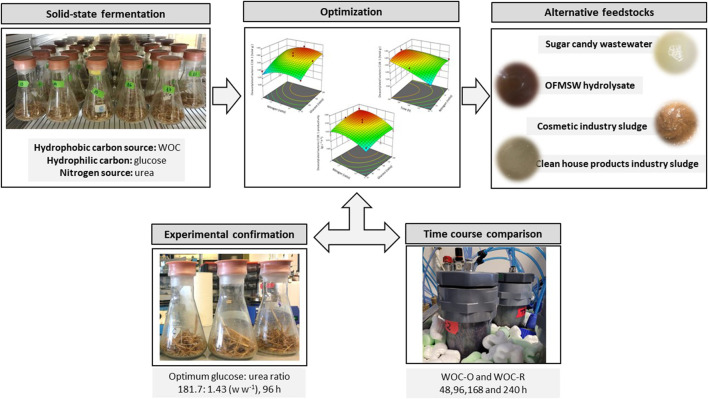
The graphical abstract of this study.

## 1 Introduction

Microbial biosurfactants (BSs) are secondary metabolites proposed as potential substitutes for chemical surfactants due to their flexibility in a wide range of environmental conditions, biodegradability, low toxicity, and ecofriendly characteristics (Andersen et al., 2016). Recent studies indicate that BSs have potential applications in the biomedical field, specifically for drug delivery and as biocidal agents against viruses such as SARS-CoV-2 (Daverey et al., 2021). Moreover, in the environmental field, sophorolipids (SLs), rhamnolipids, and lipopeptides have emerged as the main BS applied for enhancing agricultural practices and improving soil quality (Eras-Muñoz et al., 2022). From an economic point of view, the global market size of chemical surfactants is forecasted to achieve a compound annual growth rate (CAGR) of 5.3% from 2020 to 2027. Similarly, BSs are expected to experience a CAGR of over 5.5% between 2020 and 2026 (Pardhi et al., 2022). In this context, SLs exhibit significant potential due to the high productivity levels of the wild-type producer *Starmerella bombicola* ATCC 2214 and the possibility of producing target congeners using engineered strains (Van Bogaert et al., 2016; Dierickx et al., 2022).

According to the literature, SLs are produced under nitrogen limitation when the microorganism reaches the stationary growth phase (Roelants et al., 2019; Wang et al., 2019). When there is an excess of a nitrogen source in the media, nutrients are used for microorganism growth and maintenance; consequently, SL production decreases (Ingham and Winterburn, 2022). It is well known that yeast growth is nitrogen dependent since it affects the formation of biomass, which in turn affects the duration and kinetics of the fermentation process (Ma et al., 2011; Christofi et al., 2022). To increase SL production, particular efforts have been made to optimize nutrient concentrations such as the nitrogen source, hydrophilic carbon source, and hydrophobic carbon source (Shah et al., 2017; Jadhav et al., 2019; Van Renterghem et al., 2019). Studies conducted on this topic agree that the simultaneous addition of both carbon sources strongly stimulated SL production. However, if the fermentation media contain only one of these sources, the growth and yeast metabolism are affected, making the process inefficient (Liu et al., 2021). Gao et al. (2013) contradicted this theory by reporting that higher production yields (>200 g L^-1^ day^-1^) can be achieved when supplementing the hydrophobic source during the yeast stationary phase. An effective substrate combination that favors SL synthesis is constituted by glucose often combined with a hydrophobic carbon source rich in oleic acid (Van Bogaert et al., 2016; Wongsirichot et al., 2021). When this source is a triglyceride, it is first converted into fatty acids by enzymes such as aldehyde dehydrogenase or long-chain alcohol oxidase, and then, they are used for SL biosynthesis, typically composed of a fatty acid chain with approximately 16–18 carbon atoms (Intasit and Soontorngun, 2023). In addition, nitrogen is an essential source that needs to be well-balanced to allow growth and reach the stationary growth phase for an optimal process (Albrecht et al., 1996; Wongsirichot et al., 2022). In recent times, there has been a concerted effort to reduce production costs and enhance the economic competitiveness of SLs. To achieve this objective, significant attention has been directed toward the utilization of alternative feedstocks, non-food competition, and the revalorization of food waste (Jiménez-Peñalver et al., 2019; Kaur et al., 2019; Wongsirichot et al., 2022).

BSs are traditionally produced via submerged fermentation (SmF). However, solid-state fermentation (SSF) seems to be a promising technology to increase efforts toward a circular economy. SSF is developed in the absence or limitation of free water (Pandey, 2003) and allows the sustainable conversion of organic insoluble solid waste into high-value-added products (Jiménez-Peñalver et al., 2016; Hu et al., 2021). The main challenges of SSF revolve around sample heterogenicity and mass and heat transfer, which are intrinsic characteristics of solid matrices (Kumar et al., 2021; Oiza et al., 2022). Temperature and composition gradients are often reported when scaling up SSF systems, pointing to the co-existence of different metabolic states for cells growing in the solid matrix. The production of SLs by SSF has been already proven feasible at different operation scales up to 100 L (Rodríguez et al., 2021). However, the nitrogen and carbon dynamics for sophorolipid production under SSF have not yet been specifically addressed.

To enhance our knowledge about SL production in SSF, it is crucial to evaluate the influence of nutrient sources on yeast growth, the production process, and the final product composition. In this way, a statistical design of experiments (DoE) was applied in this study. The application of DoE is widely used for biological process optimization and has already been used for biosurfactant production such as surfactin (Zhu et al., 2013) and SLs (Minucelli et al., 2017). DoE provides an understanding of the interactions between factors (medium components) at different levels (concentration/ratio) and their effect on the evaluated output. The variables that are found significant and fit a statistical model (linear, quadratic, and cubic curvature, among others) can be further optimized using the response surface methodology (RSM) that has been used extensively for media optimization (Rispoli et al., 2010). Since there are a large number of possible combinations to be tested when DoE is applied, the use of bioreactors is a limitation to the analysis being the shake flask scale, the methodology reported in the literature (Ingham and Winterburn, 2022).

This paper aims to evaluate the influence of hydrophilic carbon and nitrogen sources on the production of diacetylated lactonic C18:1 SLs, focusing on the variation in the glucose and urea ratio by applying a Box–Behnken design (BBD). Our hypothesis is based on balancing nitrogen to ensure suitable cell growth levels and optimal SL productivity. To the best of our knowledge, this is the first instance to explore SL production and optimize diacetylated lactonic C18:1 production on multiple substrates via SSF. Winterization oil cake (WOC), sweet candy industry wastewater, hydrolysate of the organic fraction of municipal solid waste, and sludges from the cosmetic industry were used as sources of hydrophobic carbon, hydrophilic carbon, nitrogen, and micronutrients for SL production on SSF.

## 2 Materials and methods

### 2.1 Pure substrates and support material

The substrates used for SL production were glucose as a hydrophilic substrate, urea as a nitrogen source, and yeast extract as a nutrient source, all of which were of analytical grade and provided by Sigma-Aldrich (St. Louis, MO, United States). In our study, WOC obtained from sunflower oil refining was used as the hydrophobic substrate, with an oil content ranging from 44% to 80%, composed mainly of 84% of C18:1 fatty acid, as described in previous studies (Jiménez-Peñalver et al., 2016; Rodríguez et al., 2021), and was provided by Lípidos Santiga S.A*.* (Barcelona, Spain). The organic support used for SSF was wheat straw provided by the Veterinary Faculty of Universitat Autònoma de Barcelona (Barcelona, Spain).

### 2.2 Yeast culture preparation

The yeast *S. bombicola* ATCC 22214 was purchased from the American Type Culture Collection (Manassas, United States) and cryopreserved at −80°C with glycerol (10% v v^−1^). It was grown for 48 h at 30 °C on agar plates containing 10 g L^-1^ of dextrose, 5 g L^-1^ of peptone, 3 g L^-1^ of malt extract, 3 g L^-1^ of yeast extract, and 20 g L^-1^ of bacteriological agar. Then, it was transferred to 100 mL broth in a 500-mL Erlenmeyer flask with the same medium composition as described above but without bacterial agar. Next, it was incubated to a mid-exponential growth in a shaker at 180 rpm for 48 h at 30 °C, reaching an absorbance reading around 0.1–0.35 with a target OD_600_ of 12–15 units (approximately 10^9^ CFU mL^-1^).

### 2.3 Solid-state fermentation

The experiments were carried out using 0.5-L Erlenmeyer flasks, with a total working volume of 0.18 L. The total solid substrate weight was approximately 20.3 ± 0.6 g with a dry matter of 53.5% (10.84 g). Each sample was loaded with a solid matrix made up of 3.8 g of wheat straw as the support, working at 75% water holding capacity and 6.3 g of WOC. The final production mixture was 43.1% aqueous phase composed of 7 mL of a nutrient dilution mix (glucose, yeast extract, and urea) and 1.7 mL of *S. bombicola* inoculum. This mixture was based on previous works using 0.1 g glucose g^-1^ dry matter as an initial stage for SL production (Jiménez-Peñalver et al., 2016; Rodríguez et al., 2020). Moreover, nutrients were added following a glucose:yeast extract:urea ratio of 100:10:01 (w w^−1^ based on the initial mixture dry weight), as extensively reported for SmF. This ratio was modified according to the experimental design. Fermentations were carried out under sterile conditions, for which wheat straw was autoclaved (121°C, 30 min) twice before the preparation of the solid matrix, and then, the total mixture was autoclaved (the same conditions) before assembly.

### 2.4 Experimental design

The Design-Expert 12^®^ program (Stat-Ease, Inc., United States) was used to generate a DoE. Glucose, urea, and fermentation time were chosen as factors and tested at three different levels, namely, low, medium, and high (Table 1). As outcomes, diacetylated lactonic C18:1 production (total g), diacetylated lactonic C18:1 productivity (g L^-1^ h^-1^) reported by working volume (0.18 L), and yeast growth (total CFU) were evaluated using a BBD (Box and Behnken, 1960). The total setup consisted of 33 runs setting a triplicate in the central point for each fermentation time for pure error estimation.

**TABLE 1:** Selected factors and levels for the designed experiment and the optimization process.

Parameter	Unit	Level −1	Level 0	Level +1
Glucose	Ratio (w w^−1^)^a^	50	125	200
Urea	Ratio (w w^−1^)^a^	0	1	2
Time	Hour	96	168	240

^a^ Ratio values were calculated based on the initial solid dry weight (10.8 ± 0.6 g).

Equation 1 shows a second-order polynomial model that was fitted for each response result. The fit significance of the model equations was evaluated using the statistical analysis of variance (ANOVA) with a *p*-value below 0.05. The fit quality of the quadratic model was expressed by the coefficient of determination (*R* ^2^) and their prediction capability by the predicted *R* ^2^.

$$y=\beta o+\sum_{i=1}^{3}\beta_{i}X_{i}+\sum_{i=1}^{3}\beta_{ii}X_{i}^{2}+\sum_{i=1}^{2}\sum_{j=i+1}^{3}\beta_{ij}X_{i}X_{j},$$ (1)

where y is the predicted response, βo is the model constant, $\beta_{i}$ , $\beta_{ii}$ , and $\beta_{ij}$ are the regression coefficients of linear, quadratic, and cross-product terms, respectively, and $X_{i}$ and $X_{j}$ are coded independent variables.

Optimum ratio predictions were generated focusing on maximizing diacetylated lactonic C18:1 production and productivity. The experimental and predicted response values were compared, and the predictive capability of the model was assessed. Multiple regression analysis was applied to analyze the variables by obtaining a regression equation that could predict the response within the specified range. To verify the obtained models, the best ratio combination was assayed by triplicate using the same setup system. When required, SigmaPlot 12.5 (Systat Software Inc., United States) was implemented for graph creation and for treatment means comparison by Tukey’s and Dunnett’s tests (*p* < 0.05).

### 2.5 Time course of optimal fermentation and comparison to reference conditions

To compare the optimum glucose:nitrogen combination (WOC-O) and the reference ratio (100:1 w w^−1^, WOC-R), the process was scaled up to 0.5-L packed bed bioreactors with a working volume capacity of 75%. Each reactor was filled with a solid matrix that included 14 g of wheat straw (water-holding capacity 75%), along with 23.20 g of WOC. Furthermore, the fermented solid consisted of a 45.1% aqueous phase, comprising 25.86 mL of a nutrient solution (glucose, yeast extract, and urea) and 6.4 mL of the *S. bombicola* inoculum. Subsequently, the total weight of the solid substrate reached 77.42 g, with a dry matter content of 54.9% for the optimum and 73.89 g with a dry matter content of 52.7% for the reference mixtures. The assay was conducted using a respirometer, and four bioreactor replicates were run for each condition. The bioreactors were sacrificed for analysis at 48, 96, 168, and 240 h.

Fermentation conditions were set as previously reported by Jiménez-Peñalver et al. (2016). In brief, the temperature was kept constant at 30°C by submerging the reactors in a water bath. A flow rate of 30 mL min^-1^ was continuously supplied to the reactors with humidified air regulated by using a mass flow controller (Bronkhorst, Spain). The oxygen uptake rate (OUR) was calculated as an indirect measure of biological activity from the oxygen concentration values in the exhaust gases (Ponsá et al., 2010; Jiménez-Peñalver et al., 2016).

### 2.6 Alternative substrates

With the aim of substituting pure substrates, different organic industrial wastes were used as feedstock. Sweet candy industry wastewater (RSC) was provided by Chupa Chups S.A.U. (Barcelona, Spain). The organic fraction of municipal solid waste (OFMSW) was kindly provided by Mancomunitat La Plana (Malla, Barcelona), and the hydrolysate (ROF) was prepared as described by Molina-Peñate et al. (2022). Nitrogenous sludges (RHP and RAC) were provided by two local industries (Barcelona, Spain). The RHP sludge comes from the cleaning of the reactors used in the production of cosmetics, hair treatments and body creams, among others while RAC comes from the cleaning of the reactors employed to produce household cleaning products. Alternative substrates were characterized and kept at −20°C. Subsequently, a series of experiments were conducted at a shake flask scale at 30°C for 100 h. The alternative substrates were dosed to achieve the optimal nitrogen and carbon levels dictated by the previously obtained model. These experiments were designed based on the potential exhibited by alternative substrates such as carbon or nitrogen sources. To reach the optimal ratio conditions of the DoE, the solid substrate was supplemented with glucose and/or urea when necessary.

### 2.7 Routine analysis

#### 2.7.1 Analytical methods

Substrate physiochemical characterization parameters such as pH, dry matter (DM), moisture content (MC), and organic matter (OM) were measured according to standard methods (Thompson et al., 2001). In addition, solid–liquid water extraction was performed (1:10, w v^−1^) at 150 rpm for 30 min. Then, the extracts were filtered using a 0.45-µm membrane filter and used for glucose, total carbon (TC), and total nitrogen (TN) analysis. The YSI 2950D biochemistry analyzer was used (YSI Inc./Xylem Inc., United States) for glucose quantification, while for TC and TN analysis, the multi N/C 2100S analyzer (Analytik Jena, INYCOM, Instrumentación y Componentes, S.A, Spain) was used.

Viable cell numbers were quantified by counting colony-forming units (CFUs), as described by Rodríguez et al. (2020). To sum up, the fermented solid was mixed with Ringer^®^ sterile saline solution (1:10, w v^−1^). Then, the mixture was shaken in an orbital incubator at 200 rpm, 25°C for 20 min, and serial dilutions were carried out (1:10, v/v). Later, 100 μL of each dilution was inoculated on agar plates and incubated at 30°C for 48 h. After incubation, the formed colonies were counted using the schuett counter (Göttingen, Germany).

#### 2.7.2 Sophorolipid extraction

Solvent extraction was performed using ethyl acetate (1:10, w v^−1^), as described by Jiménez-Peñalver et al. (2018). In brief, the mix was shaken twice in an orbital incubator at 200 rpm, 25°C for 1 h. The extracts were pooled together, and anhydrous Na_2_SO_4_ was added to remove moisture traces. Next, the samples were filtered using Whatman filter paper No. 1 and vacuum-dried using a rotary evaporator at 40°C. Following this, the resulting SL crude extract was cleansed of any oily residue by washing it with n-hexane and leaving it to dry overnight. Finally, the SL crude extract was determined gravimetrically and stored at 4°C until further use in post-fermentation procedures.

#### 2.7.3 HPLC–UV quantification method

Diacetylated lactonic C18:1 was quantified following the method proposed by Ingham et al. (2023) with some modifications. The SL crude extract was diluted in ethanol (10 g L^-1^), heated at 60°C for 15 min to dissolve the lactonic SL, and filtered using a 0.22-µm membrane before analysis. Then, molecules were separated in the HPLC UltiMate™ 3000 system (Thermo Fisher Scientific, Spain) using the Nucleosil™ 100 × 3 × 4.6 mm C18 EC column (Phenomenex, United States). The method conditions were a flow rate of 1.4 mL min^-1^, column temperature of 45°C, and injection volume of 10 µL measured using a UV visible diode array detector at a spectrum of 198 nm. A solution of acetonitrile/water, both supplemented with 0.1% formic acid, was used as the mobile phase. The elution gradient was set at 70/30 for 10 min, followed by a linear gradient up to 10/90 in 50 min; this ratio was maintained for an additional 10 min, after which it was set back to 70/30 for 15 min to restore initial conditions. Moreover, with the aim of identifying the compounds by their mass/charge (m/z), samples after the LC–UV were ionized by electrospray (in the negative mode) and were analyzed using a MicroTOF-Q II mass spectrometer (Bruker, United States) coupled to the equipment. Finally, the calibration curve concentration ranged from 2.5 to 20 g L^-1^ using the standard 1′,4″-sophorolactone 6′,6″-diacetate with a purification of ≥80% (Cayman Chemical, United States).

## 3 Results and discussion

As mentioned earlier, the evaluation of glucose as a hydrophilic carbon source and urea as a nitrogen source was performed based on their weight ratio related to the initial total mixture dry weight. To screen potential alternative feedstocks for SL production on SSF, a deep understanding of the media components and product range, as well as potential interactions, is required. In this context, Table 2 shows the BBD matrix outcomes for the analyzed responses.

**TABLE 2:** Box–Behnken design matrix and observed responses: production and productivity of diacetylated lactonic C18:1 and yeast growth.

Run	Combination	$X_{1}:glucose$	$X_{2}:urea$	$X_{3}:time$	Diacetylated lactonic C18:1 production		Diacetylated lactonic C18:1 productivity	Yeast growth
		(Ratio)	(Ratio)	(h)	(Total g)	(g g^-1^ DM_i_)	(g L^-1^ h^-1^)	(Total CFU)
1	+++	200	2	240	0.624	0.054	0.014	4.57 × 10^10^
2	-++	50	2	240	0.408	0.040	0.009	3.90 × 10^10^
3	-0-	50	1	96	0.396	0.039	0.023	2.53 × 10^10^
4	+00	200	1	168	0.736	0.062	0.024	3.36 × 10^10^
5	0--	125	0	96	0.242	0.022	0.014	5.76 × 10^10^
6	+0+	200	1	240	0.866	0.073	0.02	3.45 × 10^10^
7	0-+	125	0	240	0.735	0.067	0.017	3.94 × 10^10^
8	--+	50	0	240	0.44	0.043	0.01	4.82 × 10^10^
9	000^a^	125	1	168	0.771	0.070	0.025	2.24 × 10^10^
10	0–0	125	0	168	0.45	0.041	0.015	3.22 × 10^10^
11	+0-	200	1	96	0.534	0.045	0.031	4.08 × 10^10^
12	+--	200	0	96	0.333	0.028	0.019	3.97 × 10^10^
13	++-	200	2	96	0.597	0.050	0.035	4.59 × 10^10^
14	-+0	50	2	168	0.409	0.040	0.014	2.08 × 10^10^
15	---	50	0	96	0.329	0.032	0.019	5.45 × 10^10^
16	−0+	50	1	240	0.438	0.043	0.010	2.32 × 10^10^
17	+-+	200	0	240	0.699	0.059	0.016	4.37 × 10^10^
18	000^b^	125	1	168	0.73	0.067	0.024	5.48 × 10^10^
19	000^c^	125	1	168	0.742	0.068	0.025	5.31 × 10^10^
20	0 + 0	125	2	168	0.531	0.048	0.018	7.93 × 10^10^
21	00+^a^	125	1	240	0.763	0.070	0.018	4.39 × 10^10^
22	++0	200	2	168	0.626	0.053	0.021	3.78 × 10^10^
23	- + -	50	2	96	0.311	0.030	0.018	3.07 × 10^10^
24	--0	50	0	168	0.337	0.033	0.011	2.98 × 10^10^
25	00-^a^	125	1	96	0.591	0.054	0.034	3.59 × 10^10^
26	−00	50	1	168	0.338	0.033	0.011	2.50 × 10^10^
27	00-^b^	125	1	96	0.587	0.053	0.034	2.78 × 10^10^
28	00-^c^	125	1	96	0.537	0.035	0.031	4.99 × 10^10^
29	0+-	125	2	96	0.529	0.048	0.031	2.58 × 10^10^
30	+-0	200	0	168	0.685	0.058	0.023	6.53 × 10^10^
31	00 + ^b^	125	1	240	0.617	0.056	0.014	8.53 × 10^10^
32	00 + ^c^	125	1	240	0.838	0.076	0.019	5.70 × 10^10^
33	0++	125	2	240	0.528	0.048	0.012	4.57 × 10^10^

^*a*^ DM, initial dry matter; ^*, b, c*^ are biological replicates at the central point for the corresponding fermentation time.

### 3.1 Nutrient influence on sophorolipid production

**FIGURE 1:**
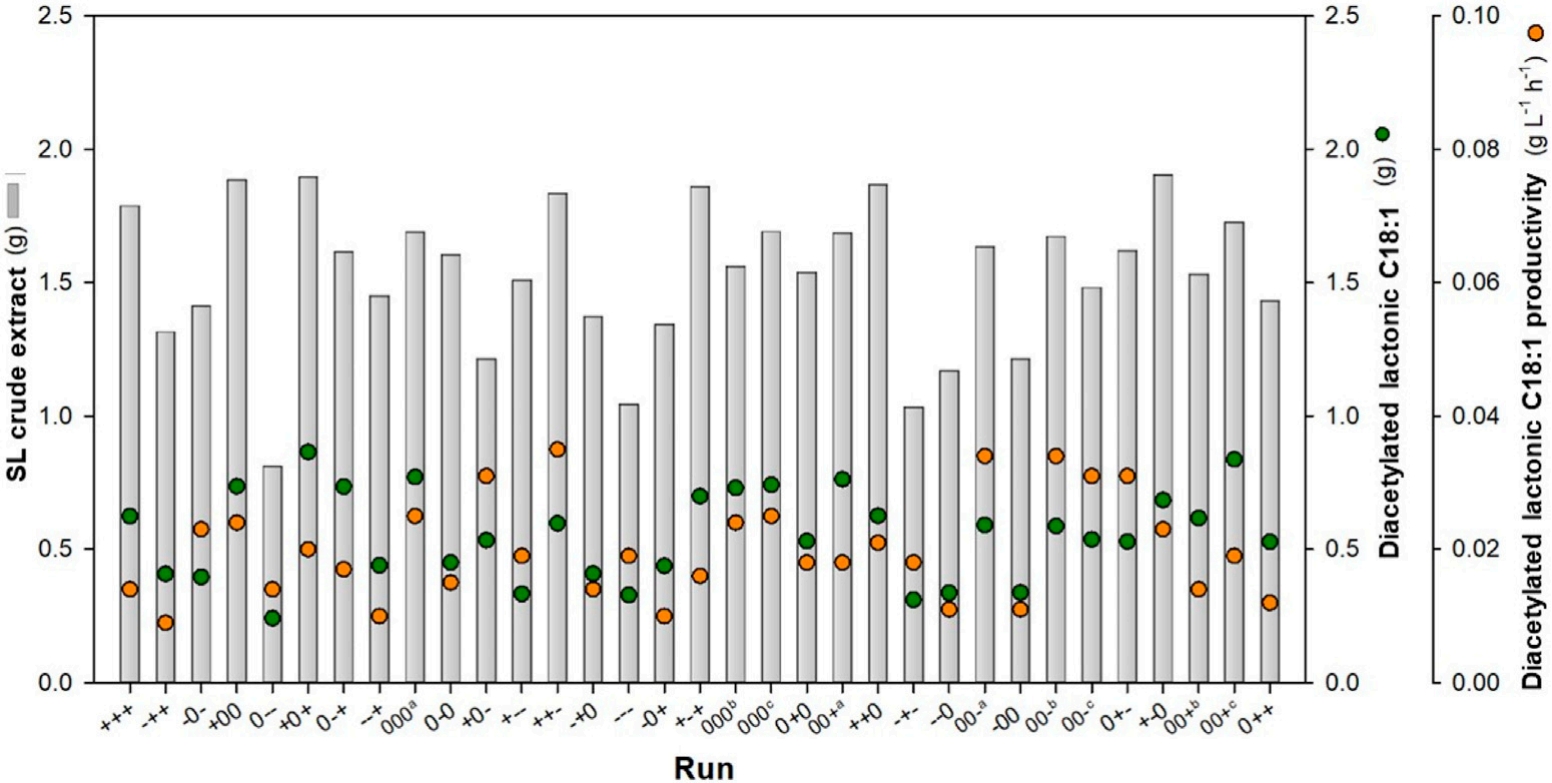
DoE outcomes based on the glucose:nitrogen ratio and fermentation time. Total gram of crude SL and C18:1 was obtained from the total initial solid wet weight (20.3 ± 0.63 g). Productivity values are expressed in working volume (0.18 L).^a, b, c^ show central point replicates.

The applied levels of glucose and nitrogen and time yielded a range of diacetylated lactonic C18:1 quantities at harvest from 0.242 to 0.866 total gram, which corresponds to a yield from 0.022 to 0.073 g g^-1^ DM_i_ (Figure 1). The central point 000 (runs 9, 18, and 19) combined with a glucose/urea weight ratio of 125:1 at 168 h resulted in a mean of 0.748 ± 0.021 total gram of diacetylated lactonic C18:1, with a yield of 0.068 ± 0.002 g g^-1^ DM_i_ and a volumetric productivity of 0.025 ± 0.001 g L^-1^ h^-1^. Moreover, the highest value of diacetylated lactonic C18:1 was achieved when a glucose/urea ratio of 200:1 was applied at 240 h (+0+). In contrast, the minimum value was achieved at 96 h, related to the combination of glucose/urea ratio of 125:0 (0--). Glucose would be depleted in the first 96 h in the combinations with a lower initial glucose ratio, showing a statistical decrease in SL production, while high ratios increased productivity, which is aligned with the results obtained by Ingham and Winterburn (2022).

Considering the importance of time as a critical operation factor, the results were assessed at each fermentation time, recognizing that the duration required to reach the stationary phase is dependent on the medium concentration (Gao et al., 2013). The results revealed that runs 13 (++-, 0.597 total g), 9 (000, 0.771 total g), and 6 (+0+, 0.866 total g) exhibited the highest production levels at 96, 168, and 240 h, respectively. In addition, the highest productivity was achieved at 96 h by run 13 (++-), achieving 0.035 g L^-1^ h^-1^. SL crude extract production increased from 0.810 to 1.905 total g, with a yield range between 0.075 and 0.164 g g^-1^ DM_i_. The literature shows that glucose is an important parameter for the SL structure. When glucose is supplied, together with a hydrophobic carbon source, it is directly incorporated into the SL, although glucose is not taken from the fatty acid synthesis. In contrast, when the glucose concentration is low, part of the fatty acids will be used for cell maintenance rather than SL synthesis since part of the fatty acids will be directed toward the β-oxidation (Hommel et al., 1994; Van Bogaert et al., 2007; Van Bogaert et al., 2016).

SL production has been extensively reported based on the crude extract, both in SmF and SSF. Compared to the extraction of SLs from liquid matrices, downstream processing in SSF presents notable differences, primarily attributed to the unique characteristics of the involved solid fermented matrix, which can decrease the efficiency of the hexane extraction process. Consequently, impurities can be present in the final crude product, affecting the downstream process design and overall economic performance. Martínez et al. (2022) described SL recovery from the solid matrix as the major contributor to operating costs.

In this case, diacetylated lactonic C18:1 SL accounted for 22%–49% of the total crude extract. The literature reports that several enzymes are involved in the SL metabolic pathway, allowing a mixture of more than 20 molecules. This SL mixture can be classified into acidic and lactonic, the diacetylated lactonic C18:1 being the main molecule produced by *S. bombicola* (Van Bogaert et al., 2016; Roelants et al., 2019; Liu et al., 2020). In the experiments shown herein, other SL congeners were also produced (Supplementary Figure S1). Our findings are consistent with those reported by Jiménez Peñalver et al. (2020), who documented an SL crude mixture from SSF primarily composed of diacetylated lactonic C18:1. Thus, when considering the total equivalent area of SL congeners present in the crude extract, the total relative abundance of this congener could be estimated as 56%–71% (Table 3). In contrast to the partially purified yellowish honey-like viscous product typically described in the literature and obtained in this study, pure SL exhibits a colorless appearance and transforms into a white powder when completely dried (Claus and Van Bogaert, 2017; Jiménez-Peñalver et al., 2020; Kashif et al., 2022). This honey-like texture could indicate that the extract still contains impurities such as long-chain fatty acids (LCFAs) originating from the WOC during fermentation. As the fermentation proceeds, higher SL titers and lower LCFA concentrations are observed, thus increasing the purity of crude extracts (Supplementary Figure S1).

**TABLE 3:** Production results based on SL crude extract LC–UV quantification.

Run	Combination	Crude extract		Total SL in the crude mix	Relative abundance of diacetylated lactonic C18:1 over total SL
		(Total g)	(g g^−1^ DM_i_)	(g g^-1^)	(%)^a^
1	+++	1.788	0.153	0.628	55.56
2	-++	1.315	0.131	0.518	59.93
3	-0-	1.412	0.141	0.460	60.92
4	+00	1.885	0.162	0.638	61.17
5	0--	0.810	0.075	0.495	60.40
6	+0+	1.895	0.163	0.709	64.50
7	0-+	1.614	0.149	0.660	68.97
8	--+	1.451	0.145	0.454	66.88
9	000^a^	1.690	0.156	0.679	67.18
10	0–0	1.604	0.148	0.445	62.99
11	+0-	1.213	0.104	0.648	67.99
12	+--	1.509	0.130	0.317	69.61
13	++-	1.834	0.157	0.488	66.68
14	-+0	1.372	0.137	0.458	65.09
15	---	1.044	0.104	0.447	70.54
16	−0+	1.343	0.134	0.505	64.50
17	+-+	1.860	0.160	0.670	56.09
18	000^b^	1.561	0.144	0.673	69.52
19	000^c^	1.691	0.156	0.640	68.62
20	0 + 0	1.540	0.142	0.529	65.18
21	00+^a^	1.687	0.156	0.689	65.59
22	++0	1.866	0.160	0.595	56.42
23	- + -	1.034	0.103	0.508	59.24
24	--0	1.170	0.117	0.431	66.97
25	00-^a^	1.634	0.151	0.535	67.63
26	−00	1.214	0.121	0.406	68.62
27	00-^b^	1.673	0.154	0.500	70.19
28	00-^c^	1.481	0.137	0.367	71.43
29	0+-	1.620	0.149	0.466	70.08
30	+-0	1.905	0.164	0.522	68.85
31	00 + ^b^	1.532	0.141	0.615	65.42
32	00 + ^c^	1.726	0.159	0.709	68.51
33	0++	1.431	0.132	0.591	62.43

^a^ Percentage values were calculated based on the SL relative area on the crude extract. DM_i_, initial dry matter.

^a,b,c^ are biological replicates at the central point for the corresponding fermentation time.

### 3.2 Diacetylated lactonic C18:1 production and productivity models and optimization

The data obtained from the fermentation processes were analyzed using Design-Expert 12^®^ software. The report of the diacetylated lactonic C18:1 measure was used in the design to clarify the process and the influence of the analyzed factors. Table 4 summarizes the ANOVA for the main responses used for the optimization process. The lack of fit was not significant (*p* > 0.05), which is reliable in terms of prediction, showing that the variation between replicates is acceptable (Haber and Runyon, 1973). The statistical analysis for diacetylated lactonic C18:1 response and productivity resulted in a second-order polynomial approach (*p*-value <0.0001 in both outcomes), with glucose ( $X_{1\;}$ ) and fermentation time ( $X_{3\;}$ ) being the most significant parameters in terms of the *p*-value. As reported by Haber and Runyon (1973), the smaller the *p*-value, the more significant the corresponding coefficient.

**TABLE 4:** ANOVA for the surface quadratic model when diacetylated lactonic C18:1 production and productivity were used as outcomes.

		Diacetylated lactonic C18:1 production (*R* ^2^ = 84.31%)		Diacetylated lactonic C18:1 productivity (*R* ^2^ = 86.23%)	
Source	DF	Mean square	*p*-value	Mean square	*p*-value
**Model**	9	0.0856	<0.0001^a^	0.0002	<0.0001^a^
$X_{1\;}$ -glucose	1	0.2923	<0.0001^a^	0.0003	<0.0001^a^
$X_{2\;}$ -nitrogen	1	0.0054	0.3610	0.0000	0.0679
$X_{3\;}$ -time	1	0.1765	<0.0001^a^	0.0007	<0.0001^a^
$X_{1\;}X_{2\;}$	1	0.0010	0.6952	0.0000	0.3424
${X_{1\;}X}_{3\;}$	1	0.0189	0.0951	1.218 × 10^−6^	0.7394
$X_{2\;}X_{3\;}$	1	0.0597	0.0051^a^	0.0001	0.0028^a^
${X_{1}}^{2}$	1	0.0416	0.0166^a^	0.0000	0.0432^a^
${X_{2}}^{2}$	1	0.1145	0.0003^a^	0.0002	0.0006^a^
${X_{3}}^{2}$	1	0.0090	0.2420	0.0000	0.2844
Residual	23	0.0062		0.0000	
Lack of fit	17	0.0068	0.3377	0.0000	0.0513

^a^ Significant parameters (*p* < 0.05).

Bold values represent parameters interaction.

Regarding diacetylated lactonic C18:1 production, the standard least squares regression (*R* ^2^) could explain 84.31% of the variability present in the analyzed response. Moreover, the model based on the predicted *R* ^2^ can also explain 64.57% of the variations in new observations, which is in reasonable agreement with the adjusted *R* ^2^ of 78.18% (difference less than 0.2). Despite the main influencing parameters, the term $X_{2\;}X_{3}$ (nitrogen and time) had a significant effect on C18:1 production when the maximum glucose ratio was applied, demonstrating a quadratic curvature and an interaction between both terms. The importance of this interaction is supported since these parameters are related to microorganism growth and survival. Finally, the resulting normalized regression produced by the model for diacetylated lactonic C18:1 production is presented in the following equation:

$$Diacetylated\;lactonic\;C18:1\;\left(total\;g\right)=0.6852+0.1274X_{1}+0.0173X_{2}+0.0896X_{3}+0.0090X_{1}X_{2}+0.0397X_{1}X_{3}-0.0705X_{2}X_{3}-0.0740{X_{1}}^{2}-0.1227{X_{2}}^{2}-0.0350{X_{3}}^{2},$$ (2)

where $X_{1}$ represents the glucose ratio, $X_{2}$ represents the nitrogen ratio, and $X_{3}$ represents the fermentation time (h).

For productivity response, *R* ^2^ was of 86.23% with an adjusted and predicted *R* ^2^ of 80.84% and 67.34%, respectively. The ANOVA shows the same behavior as the diacetylated lactonic C18:1 outcome. The regression equation for the normalized data was as follows:

$$Productivity\;\left(g\;L^{-1}h^{-1}\right)=00231+0.1274X_{1}+0.0173X_{2}+0.0896X_{3}+0.0090X_{1}X_{2}+0.0397X_{1}X_{3}-0.0705X_{2}X_{3}-0.0740{X_{1}}^{2}-0.1227{X_{2}}^{2}-0.0350{X_{3}}^{2},$$ (3)

where $X_{1}$ , $X_{2}$ , and $X_{3}$ are the glucose ratio, nitrogen ratio, and fermentation time (h), respectively.

**FIGURE 2:**
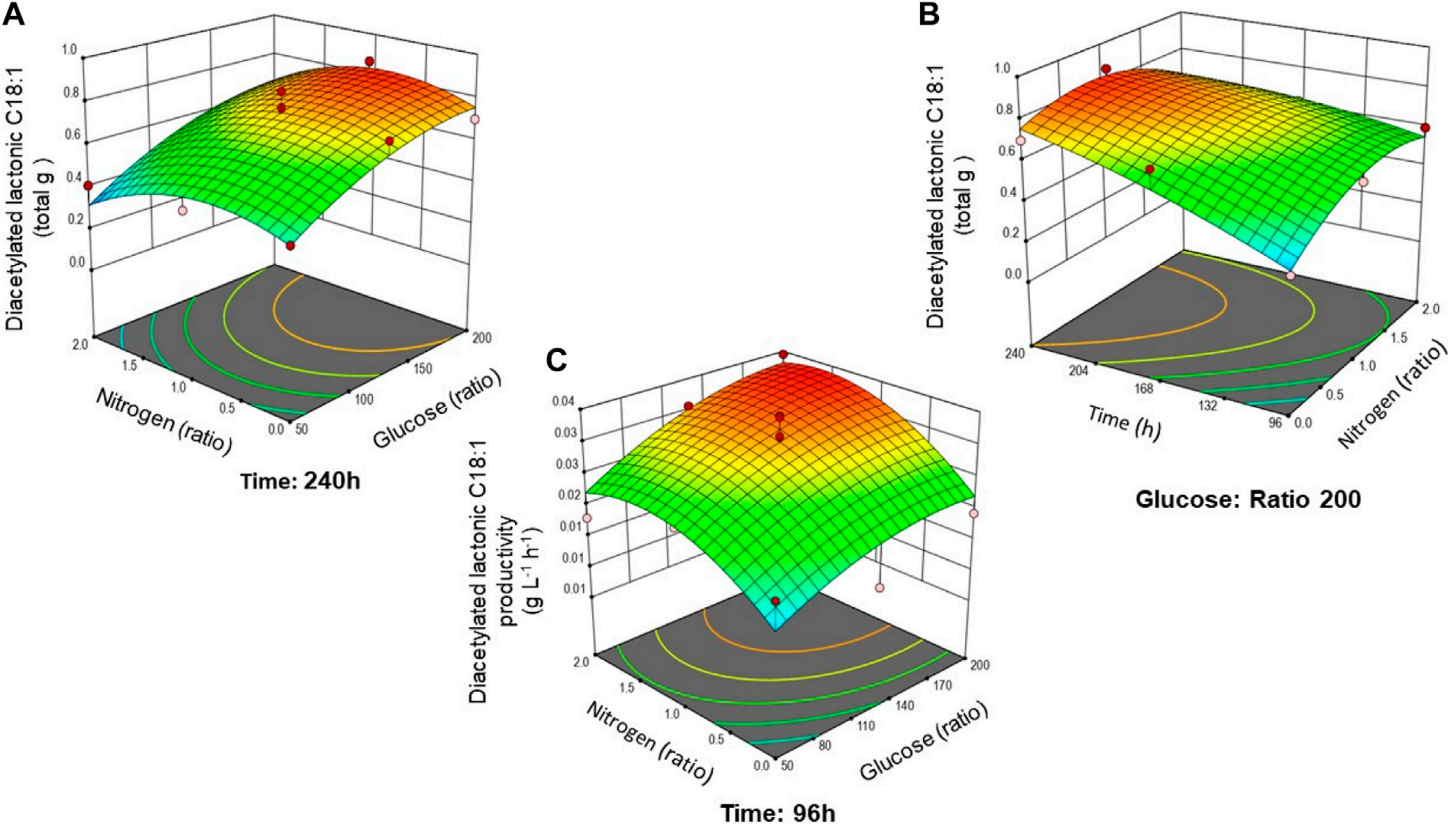
Combined effect of analyzed factors on diacetylated lactonic C18:1 production response: **(A)** glucose and nitrogen ratio at 240 h; **(B)** time and nitrogen at a maximum glucose ratio of 200; and **(C)** productivity response at 96 h based on the glucose and nitrogen ratio. Surface plots are colored from low (blue) to high (red).

To demonstrate the interaction between hydrophilic carbon (glucose) and nitrogen and fermentation time, surface plots in 3D were generated to show their effect on diacetylated lactonic C18:1 production and productivity (Figure 2). When the glucose:nitrogen ratio is modified, changes in the shape and contour of the RSM can be analyzed. Figure 2A shows that at the maximum fermentation time (240 h), a high glucose ratio (between 186.5 and 199.0) promotes the production of the lactonic SL. Nitrogen causes a lower impact compared to glucose, as can be deduced by the model parameters. Moreover, when the highest glucose ratio was set, the interaction between time and nitrogen was demonstrated (Figure 2B). The highest productivity was achieved at 96 h and decreased afterward. Figure 2C presents productivity evolution at 96 h, showing that maximum productivity was obtained when nitrogen and glucose ratios were at the highest values.

As reported by the literature, medium optimization and experimental designs for SL production in liquid cultures have been carried out by several research groups (Casas and García-Ochoa, 1999; Saerens et al., 2009; Rispoli, et al., 2010; Parekh and Pandit, 2012). Nevertheless, the scarcity of studies focused on optimizing nutrients in SSF has hindered meaningful comparisons within the field. Ingham and Winterburn (2022) developed a central composite experimental design to understand how nitrogen, glucose, and oil sources influence sophorolipid production via SmF. Their findings support that nitrogen and oil were significant, but glucose did not demonstrate a significant effect on SL production in the analyzed concentration range (15.9–184 g L^–1^). In contrast, our research demonstrated the significant influence of glucose on the production of diacetylated lactonic C18:1. These findings are aligned with those of the study conducted by Minucelli et al. (2017), who reported a reduction of 83% in SL production when the glucose concentration was decreased from 100 g L^-1^–10 g L^-1^. In addition, in our experiment, the hydrophobic carbon source was kept constant, which can also show the effect of glucose in the process.

### 3.3 Experimental validation of the optimized conditions

**FIGURE 3:**
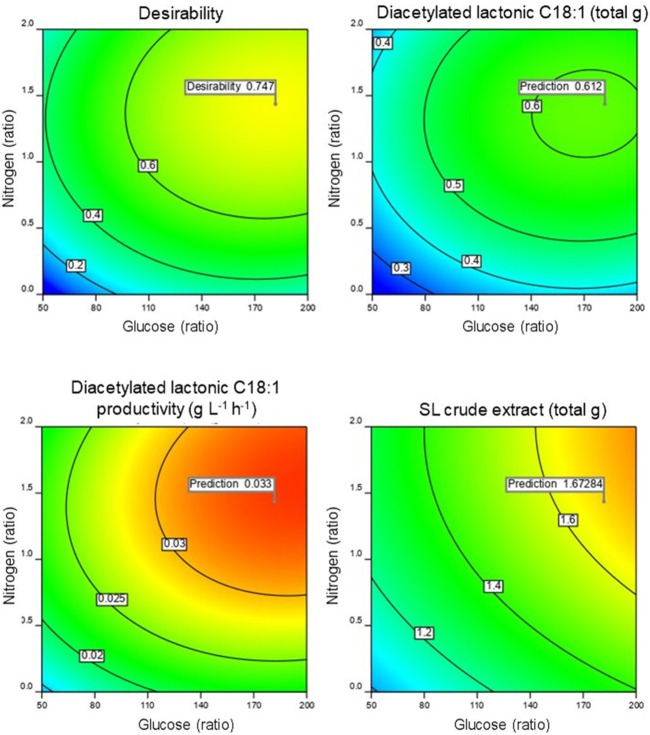
Contour profile of the predicted optimized point obtained with the achieved model.

Utilizing the acquired model, the production of diacetylated lactonic C18:1 was optimized using a numerical method provided by Design-Expert 12^®^ software. Considering the associated costs of extended fermentation time, productivity becomes a crucial factor for prospective industrial process implementation and scale-up. Therefore, the objective was to maximize lactonic SL production and productivity regardless of the yeast growth and SL crude extract. The optimal point was a glucose:nitrogen ratio of 181.75:1.43 (w w^−1^) that corresponds to a glucose and nitrogen concentration of 94 and 0.74 g kg^-1^, respectively, of the wet mixture initial weight. The optimal fermentation time was 100 h with a prediction of 0.612 total g of diacetylated lactonic C18:1 and a productivity of 0.033 g L^-1^ h^-1^ (Figure 3). It is worth mentioning that the optimal glucose ratio obtained in our study is comparable to that reported for SmF (50–100 g L^-1^). While SSF offers the advantage of significantly reduced water volumes, it is essential to acknowledge that mass transfer limitations can affect the availability of nutrients for microorganisms (Kumar et al., 2021; Chilakamarry et al., 2022; Al-Kashef et al., 2023).

**FIGURE 4:**
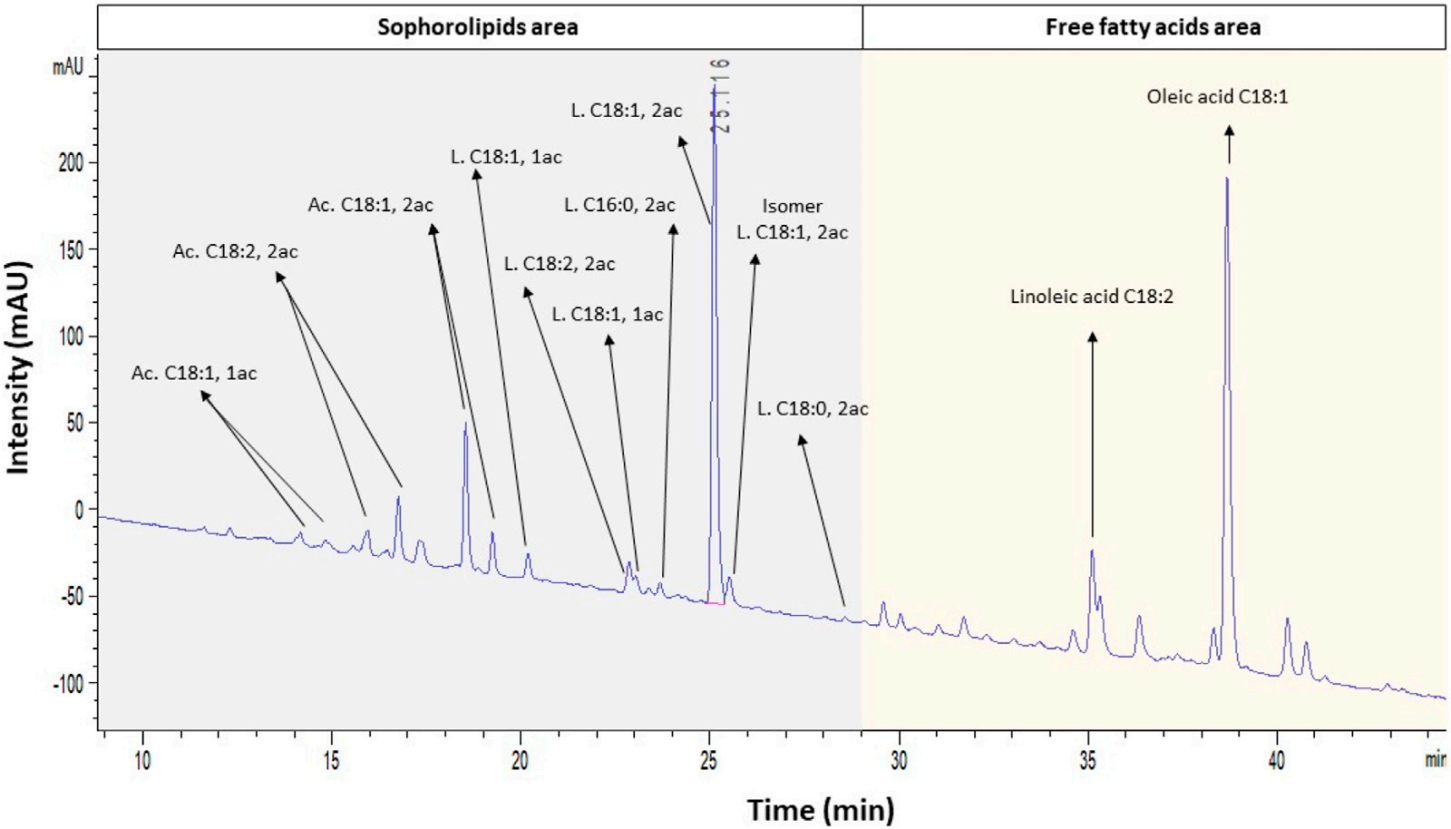
LC–UV spectra at 198 nm under the optimized condition (DLA). Identification of SLs and other compounds was developed based on LC–MS results. Ac, acidic; L, lactonic; and ac, acetylation.

The optimal conditions (DLA) were validated experimentally and tested in triplicate. The obtained results were 0.535 ± 0.007 total gram of diacetylated lactonic C18:1 SL, which corresponds to 0.047 ± 0.001 g g^-1^ DM_i_, with a productivity of 0.030 ± 0.001 g L^-1^ h^-1^. This result fits in the 95% confidence interval. As before, LC–UV analysis of the SL crude extract showed that other SL congeners were present in the crude extract mix (Figure 4). The DLA combination yielded 1.617 ± 0.031 total gram of SL crude extract, with a total SL area equivalent to 0.42 ± 0.031 g g^-1^ crude extract being the most representative congener of the diacetylated lactonic C18:1 by 76.9%.

### 3.4 Time course comparison of optimized and reference conditions in a 0.5-L packed bed bioreactor

**FIGURE 5:**
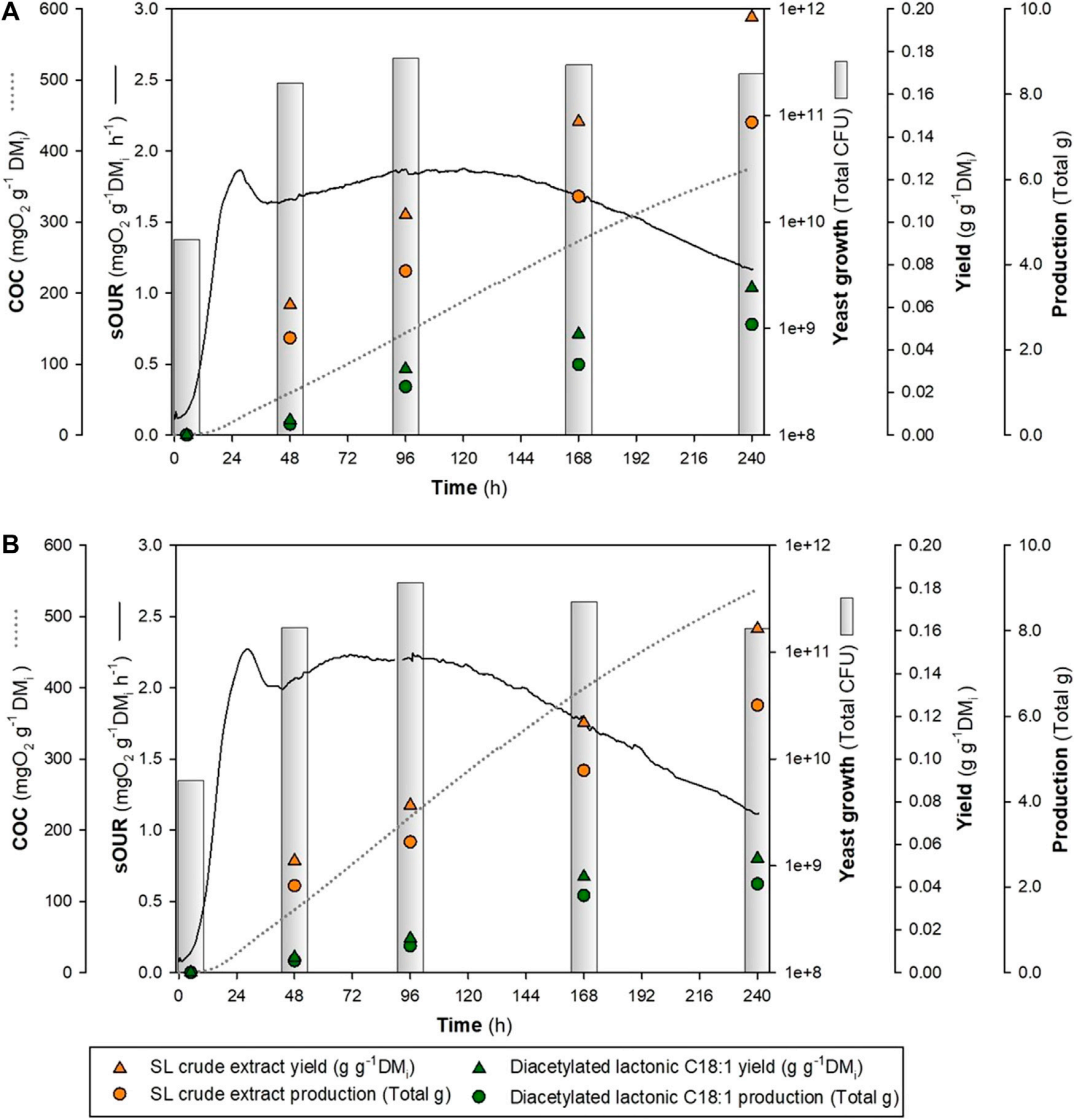
Time course OUR profile of different glucose:nitrogen ratios at 240 h in a 0.5-L reactor. **(A)** WOC-O. **(B)** WOC-R.

Similar fermentation profiles, as shown in Figure 5, were analyzed over 48, 96, 168, and 240 h at a 0.5-L reactor scale. The outcomes revealed the presence of an SL (crude extract and diacetylated lactonic C18:1) from the initial sampling at 48 h in both combinations. After fermentation (240 h), a maximum SL crude extract of 0.196 and 0.161 g g^-1^ DM_i_ was achieved by WOC-O and WOC-R, respectively. In addition, diacetylated lactonic C18:1 showed differences between both treatments with values of 0.069 g g^-1^ DM_i_ and 0.053 g g^-1^ DM_i_, respectively. These findings highlighted that WOC-O produced a 22% increase in the SL crude extract and a 30% increase in diacetylated lactonic C18:1 production compared to WOC-R. Moreover, WOC-O presented the highest volumetric productivity of the SL crude extract and diacetylated lactonic C18:1 (0.082 and 0.029 g L^-1^ h^-1^, respectively). These values are higher than those reported by Jiménez-Peñalver et al. (2016), who achieved an SL crude extract yield of 0.179 g g^-1^ DM_i_ after 240 h using WOC and sugar beet molasses as substrates.

The HPLC–UV analysis showed a higher total SL equivalent area in the WOC-O combination (6607.34 mAU*s) compared to WOC-R (5417.751 mAU*s), which represents a production of 0.461 and 0.365 g of SLs per gram of crude extract, respectively. In this sense, the main produced congener was diacetylated lactonic C18:1 which represents 51.5% in WOC-O and 55.9% in WOC-R of the total SL mix. Diacetylated acidic C18:1 was the second abundant congener, which presented an increase in the concentration over time, with an area ratio of 1:5 compared to diacetylated lactonic C18:1 (Supplementary Table S1 and Supplementary Figure S2).

Throughout the fermentation process, in both treatments, the total CFU exhibited a notable increase by two orders of magnitude (10^11^) when compared to the initial concentration (10^9^), which is aligned with the observations reported by Rodríguez et al. (2021). Glucose analysis showed the presence of a residual content of 0.010 g g^-1^ DM_i_, followed by its depletion in WOC-O, while for WOC-R, glucose depletion after 48 h sampling was observed. The fermentation profile revealed a maximum OUR peak achieved in both treatments at 28 h with values consistent with our previous published results (Jiménez-Peñalver et al., 2016; Rodríguez et al., 2020).

In summary, optimized glucose and nitrogen concentrations led to an SL crude extract yield of approximately 0.19 g g^-1^ DM_i_, exceeding yield values obtained under reference conditions herein and in previous publications with the same wild-type *S. bombicola* in SSF. Future research efforts may focus on exploring this optimal glucose:nitrogen ratio using fed-batch techniques to increase productivity.

### 3.5 Nitrogen source and yeast growth

As our hypothesis was based on nitrogen as a growth-limiting factor and we were attempting to understand growth-production dynamics under SSF, yeast growth was also evaluated as a response. The initial *S. bombicola* seed for this experiment was 1.89 × 10^9^ total CFU, which represents 1.7 × 10^8^ CFU g^-1^ DM_i_. Subsequently, the analyzed results showed that CFU increased one order of magnitude after fermentation. The central point (000) presented a yeast growth mean of 4.9 × 10^10^ ± 5.61 × 10^9^ total CFU (4.5 × 10^9^ ± 5.18 × 10^8^ CFU g^-1^ DM_i_), with an initial concentration of 0.649 g L^-1^ total nitrogen in the aqueous phase, which corresponds to a concentration of 0.001 g g^-1^ DM_i_ of urea. The highest growth was achieved in run 32 (00+) at 240 h (8.5 × 10^10^ ± 4.6 × 10^9^ total CFU). Nevertheless, the lowest growth was reached by combinations without the addition of urea, regardless of the glucose ratio in runs 15 (---), 10 (0–0), and 17 (+-+) at 96, 168, and 240 h, respectively (2.1 × 10^10^, 2.2 × 10^10^, and 2.3 × 10^10^ total CFU, respectively).

The statistical analysis for yeast growth response was fitted to a second-order polynomial approach with base 10 logarithm data transformation (*p*-value 0.0019) being the influencing factors, along with nitrogen (*p*-value 0.0005) and fermentation time (*p*-value 0.0005). For this response, the lack of fit was significant (*p*-value 0.0122), which deemed that the model is not reliable in terms of prediction (*R* ^2^ of 63.33%). The regression equation for the normalized data was as follows:

$${\mathrm{Log}}_{10}\left(Growth\right)=10.63+0.0009\;X_{1}+0.1121{\;X}_{2}+0.1005{\;X}_{3}+0.0211\;X_{1}X_{2}-0.0165\;X_{1}X_{3}+0.0329\;X_{2}X_{3}-0.0523\;{X_{1}}^{2}-0.0458{X_{2}}^{2}+0.0500{X_{3}}^{2},$$ (4)

where $X_{1}$ represents the glucose ratio, $X_{2}$ represents the nitrogen ratio, and $X_{3}$ represents the fermentation time (h).

Further details on this model are available in Supplementary Table S2 and Supplementary Figure S3. Although the model is not reliable in terms of prediction, some conclusions can be obtained from the observed trends. To maximize yeast growth, the optimal glucose:nitrogen ratio was 128.9:2 (w w^−1^) at 240 h, reaching 7.51 × 10^10^ total CFU and a production of 0.57 total g of diacetylated lactonic C18:1. As expected, this implies lower glucose and higher nitrogen and time than optimal values for production and productivity and leads to slightly lower total production but far lower productivity due to increased process time. As reported by Daverey and Pakshirajan (2010), decreasing the nitrogen source in the fermentation broth can result in a lower biomass concentration, thereby negatively impacting SL production. In their study, the optimum nitrogen concentration for biomass growth was 10 g L^-1^, while for SL production, it was 2 g L^-1^.

Gao et al. (2013) reported that at a flask scale, the cell density increased proportionally to the increase in standard Yeast Malt Broth used as the nitrogen source, which aligned with our findings. In contrast, the authors also emphasized that a high glucose concentration suppressed growth, which differs from our findings, where glucose was not a significant parameter for this response. In addition, Ma et al. (2011) found that the highest cell dry weight (12.90 g L^-1^) was achieved using the yeast extract and ammonium sulfate as the nitrogen source, with a concentration of 3 g L^-1^ and 4 g L^-1^, respectively. Nevertheless, it negatively influenced SL synthesis (29.75 g L^-1^) when compared with the control group of each nitrogen source (73.10 and 71.00 g L^-1^, respectively). This highlights the significant influence that accessible nitrogen can have on the process, emphasizing the importance of identifying the optimal nitrogen concentration to enhance SL production. Furthermore, these findings also imply the necessity of evaluating the influence that nitrogen source combinations can exert on the process.

From the results presented herein (Table 2), no clear relationship was observed between total growth and total diacetylated lactonic C18:1 production considering either all data or specific production times in the correlation analysis. This illustrates the complexity of dynamics under solid-state fermentation where higher cell growth does not necessarily mean higher SL production. In addition, SL production profiles obtained in batch-packed bed SSF reveal a significant production of SLs in the initial days of the process simultaneous to cell growth. This confirms that in solid heterogeneous matrices, cell growth and metabolite production are not two sequential phases, and different metabolic phases co-exist. Operation strategies allowing for matrix homogeneity should improve growth and, hence, SL production.

### 3.6 Alternative substrates for SL production in SSF

As the market demand for biosurfactants increases, the constant attempt to reduce production costs and environmental impacts in the biosurfactant industry has prompted research to focus on alternative sources to pure nutrients. The utilization of alternative substrates such as food waste, green residues, and industrial organic waste has positioned SL production within the context of a green bioeconomy, with a global biosurfactant market of over 5.52 billion by 2022, with an increasing rate of 5.5% per year (Markets and Markets, 2016; Singh et al., 2019). Due to the high interest generated around this topic, several techno-economic evaluations have been developed for biorefineries and scaled-up processes using alternative feedstocks (Wang et al., 2020; Martínez et al., 2022). As reported in the literature, glucose has been recognized as the principal hydrophilic carbon source utilized in SL industrial production. When supplemented with a hydrophobic source, nitrogen source, and nutrients, it enhances process efficiency (Baccile et al., 2017; Jiménez-Peñalver et al., 2019; Eras-Muñoz et al., 2022). Nevertheless, it is evident that the utilization of pure substrates increases production costs, environmental impacts, and even social impacts when using food crops. Therefore, the identification of potential residues as promising feedstocks assumes paramount importance for establishing a sustainable process. Moreover, Shah et al. (2017) reported that the oil composition influences the SL metabolic pathway, and the use of no-conventional hydrophobic carbon sources could stimulate the production of novel BS.

With the achieved optimal glucose:nitrogen ratio and time values, pure substrate substitution was assayed using industrial organic residues. Table 5 summarizes the characteristics of the different industrial residues used in this study. Based on residue characterization, RHP and RAC present potential to be used as a nitrogen source. However, large differences were found for pH and TN values related to their origin. RSC and ROF characteristics framed these residues as potential hydrophilic carbon sources due to their glucose content. Moreover, it is important to highlight that ROF could also be used as a nitrogen source, making it a versatile residue, as reported by Kaur et al. (2019) and To et al. (2023). Additionally, the combination RSO was tested using sweet candy wastewater as a hydrophilic carbon source and OFMSW hydrolysate as a nitrogen source.

**TABLE 5:** Main characteristics of the different industrial residues used as feedstock.

Substrate	DM (%)	MC (%)	OM (%, db)	pH	Glucose* (g L^-1^ or g kg^-1^)	TC* (g L^-1^ or g kg^-1^)	TN* (g L^-1^ or g kg^-1^)
Winterization oil cake (WOC)	91.87	8.13	44.02	6.04	n.d	10.50	0.04
Cosmetic sludge (RHP)	11.12	88.88	93.87	6.53	n.d	21.24	3.89
Clean house products sludge (RAC)	8.39	91.61	92.38	11.75	n.d	41.60	1.05
Sweet candy wastewater (RSC)	12.76	87.24	94.68	4.38	2.08	57.30	0.02
OFMSW hydrolysate (ROF)	2.01	97.99	99.37	5.20	17.03	28.70	1.20

db, dry basis; n.d, not detected. *Data units are expressed by volume or weight according to residue physical characteristics.

**FIGURE 6:**
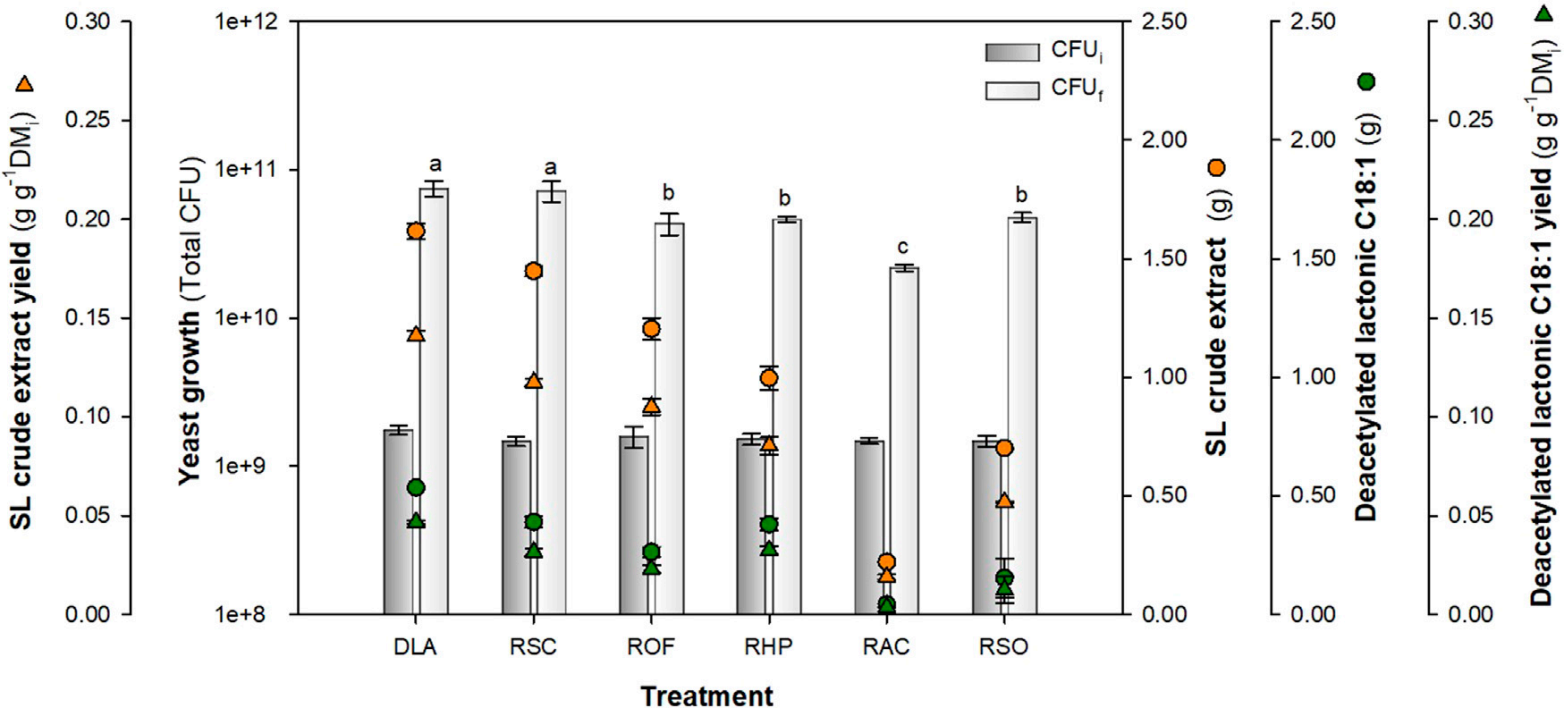
Alternative feedstocks for SL production and yeast growth at 100 h using the optimal glucose:nitrogen ratio of 181.7:1.43. Concentration values were calculated based on the working volume (0.18 L). Same letters indicate statistically insignificant differences at *p-*value <0.05 for yeast growth. DLA, control group; RSC, sweet candy wastewater; ROF, OFMSW hydrolysate; RHP, cosmetic sludge; RAC, clean house product sludge; and RSO, sweet candy wastewater + OFMSW hydrolysate.

As Figure 6 shows, the tested residues at 100 h allowed *S. bombicola* growth and SL production. The literature reported that fermentations using *S. bombicola* are associated with a pH decrease (Van Bogaert et al., 2011; Jiménez-Peñalver et al., 2018). In these experiments, pH decreased from an initial value of approximately 5.5 ± 0.25 to values of approximately 2.6–3.0 after 100 h, except for RAC which had pH of 4.6. Initial samples do not present significant differences in CFU content, as based on Tukey’s test (*p*-value >0.05). After fermentation, the control group (DLA) achieved a total yeast growth of 7.50 × 10^10^ ± 0.87 × 10^10^ CFU. Moreover, when residues were compared, the combination RSC presented the highest growth (7.24 × 10^10^ ± 1.15 × 10^10^ total CFU), while the lowest was achieved by RAC (2.19 × 10^10^ ± 0.11 × 10^10^ total CFU). Statistical Dunnett’s multiple comparison test showed that combinations ROF (*p*-value 0.0070), RHP (*p*-value 0.0118), RAC (*p*-value 0.0003), and RSO (*p*-value 0.0154) presented significant differences in yeast growth at 100 h compared with the control group.

Regarding SL production, statistical Tukey’s test showed that the highest production of diacetylated lactonic C18:1 was achieved by RSC (0.389 ± 0.024 total g) and RHP residues (0.379 ± 0.026 total g) with a productivity of approximately 0.022 ± 0.001 g L^-1^ h^-1^ (*p*-value: 0.9869). Furthermore, RAC presented the lowest production (0.043 ± 0.001 total g). When comparing residues that used OFMSW (ROF and RSO), an insignificant difference was observed (*p*-value: 0.2786). The obtained SL production results do not agree with those reported by Kaur et al. (2019), who used the OFMSW hydrolysate. For the ROF residue, the glucose present in the hydrolysate was used as a carbon source, while RSO is based on a combination of two residues: sweet candy wastewater and OFMSW hydrolysate. Although these combinations kept the evaluated nutrient ratio, it is clear that the hydrolysate also contains other type of sugars, fatty acids, and nutrients due to its provenience (Kaur et al., 2019; Pleissner and Peinemann, 2020). In this way, recent literature reported that autoclaving hydrolysates can lead to the formation of inhibitors in the media, which suggests that tangential filtration could be considered as a potential option for future investigations (Ingham et al., 2023).

In addition, SL crude extract production presented significant differences (*p*-value <0.0001) between RSC (1.448 ± 0.023 total gram with a yield of 0.117 ± 0.002 g g^-1^ DM_i_) and RHP (0.996 ± 0.051 total gram with a yield of 0.085 ± 0.004 g g^-1^ DM_i_). As mentioned before, SL congeners were present in the crude extract mix for RSC 0.38 and for RHP 0.45 g of SL per gram of crude mix, with a diacetylated lactonic C18:1 having relative areas of 71% and 84%, respectively. When SL crude extract productivity was analyzed, the best result was achieved by RSC, 0.080 ± 0.046 g L^-1^ h^-1^; this result aligned with that obtained by Rashad et al. (2014), who achieved a titer of 10 g L^-1^ with a productivity of 0.107 g L^-1^ h^-1^ on SSF.

One of the major drawbacks associated with low-cost substrates is the selection of an appropriate waste with the precise balance of carbon and nitrogen that allows significant growth and product formation. Considering the context, the obtained results demonstrate the potential of RSC as a hydrophilic carbon source and RHP as a nitrogen source for diacetylated lactonic C18:1 production. Wadekar et al. (2012) used sweet water supplemented with glycerol for SL production on SmF, achieving an SL yield of 6.36 g L^-1^ composed of 18.9% acidic SL, 19.6% lactonic C18:1, and 60.8% lactonic C18:2 SL. In the present study, the quantification results were focused on diacetylated lactonic C18:1, showing that in the optimized control group, this compound constituted 53.5% of the SL crude extract mix, while for RSC and RHP, it is around 38.9% and 37.9%, respectively. However, when the total SL area is analyzed, the diacetylated lactonic C18:1 represents 76.9% for DLA, 70.9% for RSC, and 84.4% for RHP. This suggests that the composition of the SL mixture is affected by the complex composition of residues used as the hydrophobic and nitrogen sources.

The results that focused on the residues used as nitrogen sources (RHP, RAC, and RSO) are congruent with those reported by Ma et al. (2011), who reported that inorganic N sources such as ammonium sulfate encourage the formation of acidic SL, while organic N sources promote the production of lactonic SL, which is confirmed with RHP results. The literature reports that there is a knowledge gap regarding alternative nitrogen sources for BS production (Solaiman et al., 2007; Wongsirichot et al., 2021). In this context, our research outcomes contribute positively to waste valorization in the SL production framework.

Although all residues showed a significantly lower production (*p*-value <0.05) compared to the control group (DLA), it is important to highlight that, when using residues, they contribute with glucose, nitrogen, and micronutrients, thus potentially increasing the process sustainability and reducing the amounts of pure substrates required. References that report diacetylated lactonic C18:1 yield on SSF processes could not be found in the literature. However, compared with the results obtained by Rodríguez et al. (2020), who reported a crude SL yield of 0.2 g g^-1^ DM_i_ using WOC and molasses as feedstock at 22 and 100 L, respectively, the SL yields achieved in this study are lower. Nevertheless, it should be considered that the present experiment was set up at a flask scale, and in this sense, the results could be improved in a scale-up process applying aeration and agitation (Raghavarao et al., 2003; Oiza et al., 2022).

We would like to emphasize the challenge in comparing research outcomes between SSF and SmF due to the presence of multiple differing parameters that can significantly influence production outcomes such as productivity and yield. Therefore, it is essential to acknowledge that SSF and SmF could be complementary technologies for SL industrial production when residue revalorization is the main purpose. Nevertheless, it must be highlighted that the low water and energy consumption of SSF suggests that it can be an effective and economically feasible technology for BS production.

## 4 Conclusion

To sum up, with the aim of providing knowledge about SL production through solid-state fermentation, a Box–Behnken design and response surface methodology were applied. A quadratic model was adjusted for the analyzed parameters, with glucose and time being the influencing factors for diacetylated lactonic C18:1 production and productivity, while nitrogen is the influencing factor for yeast growth, achieving the highest productivity at 100 h. A productivity of 0.033 g L^-1^ h^-1^ was achieved with a glucose:nitrogen ratio of 181:1.43 (w w^−1^ initial dry weight), reaching a yield of 0.047 g g^-1^ DM_i_ for the diacetylated lactonic C18:1 and 0.141 g g^-1^ DM_i_ for the SL crude extract. Moreover, the time course comparison in a 0.5-L packed bed bioreactor using the optimal combination showed a production increase in the SL crude extract (22%) and diacetylated lactonic C18:1 (30%) when compared with the reference medium combination. In addition, when using residues instead of pure substrates under optimal conditions, sweet candy wastewater and nitrogenous cosmetic sludge showed good potential as alternative feedstocks. Finally, due to the outcomes achieved in the time course comparison, future work on fed-batch and scale-up processes, in addition to the evaluation of alternative hydrophobic carbon source residues that can be exploited by SSF, is an open research field.

## Data Availability

The datasets for this article are publicly available in https://doi.org/10.34810/data973.
