# Supplementary material for: Carbon and nitrogen optimization in solid-state fermentation for sustainable sophorolipid production using industrial waste
Source: Front Bioeng Biotechnol. 2024 Jan 4;11:1252733. doi: 10.3389/fbioe.2023.1252733 (PMC10797751; doi:10.3389/fbioe.2023.1252733)
Supplement: Supplementary file 1 [file DataSheet1.docx]

Supplementary Material

**Carbon and Nitrogen Optimization in Solid-State Fermentation for Sustainable Sophorolipids Production Using Industrial Waste**

Estefanía Eras-Muñoz, Xavier Font and Teresa Gea*.

*** Correspondence:** [**teresa.gea@uab.cat**](mailto:teresa.gea@uab.cat)


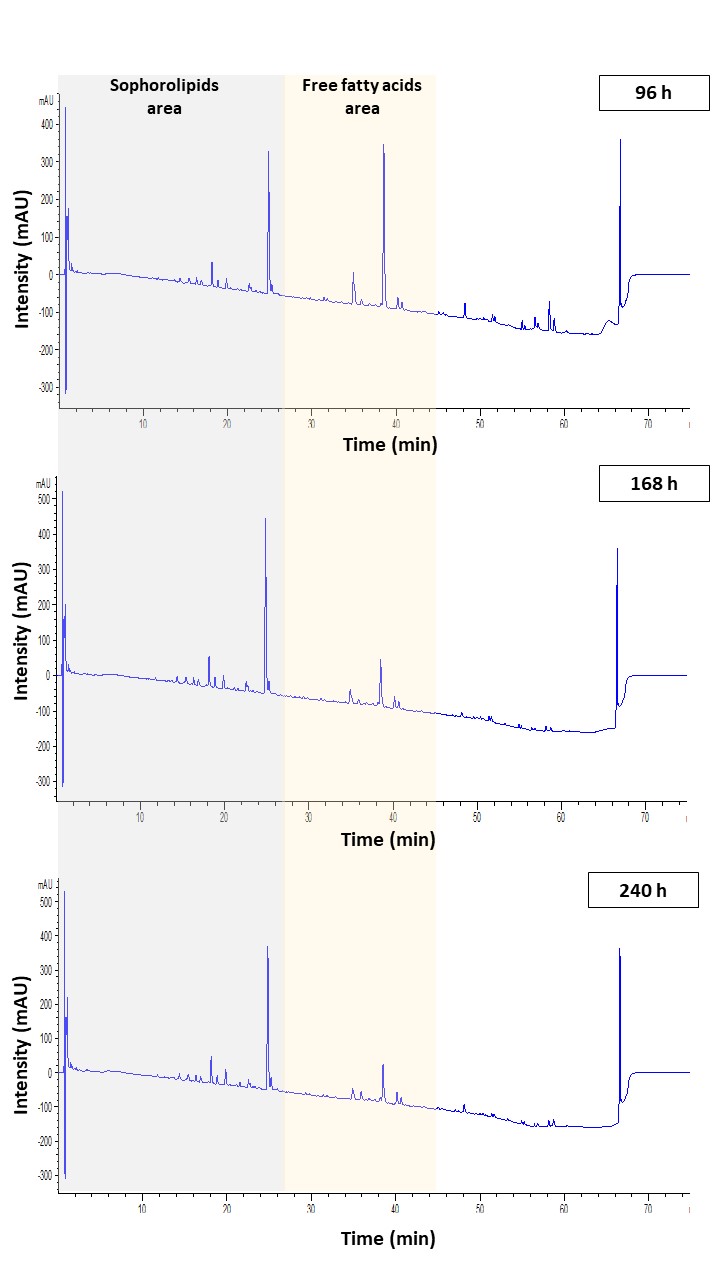


**Supplementary Figure S1.** LC-UV spectra at 198 nm. Sample belongs to the central point of the DoE with a glucose: urea ratio of 125:1 (w w^-1^) at 96, 168 and 240 h. Grey color shows sophorolipids relatively area in the crude mix while yellow represents the free fatty acids area. As can be seen fatty acids decrease proportionally with fermentation time.

**Supplementary Table S1.** Sophorolipid total area comparison between WOC-O and WOC-R.

| **Sophorolipid congeners** | **Retention time (min)** | **Area (mAU*s)** | | **Area (%)** | |
| --- | --- | --- | --- | --- | --- |
|  |  | **WOC-O** | **WOC-R** | **WOC-O** | **WOC-R** |
| Ac. C18:1, 1ac | 13.66 | 136.46 | 106.26 | 2.07 | 1.96 |
| Ac. C18:1, 1ac* | 14.33 | 75.36 | 45.91 | 1.14 | 0.85 |
| Ac. C18:2, 2ac | 15.47 | 245.50 | 177.182 | 3.72 | 3.27 |
| Ac. C18:2, 2ac* | 16.31 | 220.29 | 126.101 | 3.33 | 2.33 |
| Ac. C18:1, 2ac | 18.09 | 707.70 | 597.65 | 10.71 | 11.03 |
| Ac. C18:1, 2ac* | 18.80 | 209.22 | 173.846 | 3.17 | 3.21 |
| L. C18:1, 1ac | 19.64 | 160.48 | 113.83 | 2.43 | 2.10 |
| L.C18:2, 2ac | 22.30 | 104.69 | 88.120 | 1.58 | 1.63 |
| L. C18:1, 1ac | 22.48 | 67.72 | 58.631 | 1.03 | 1.08 |
| L. C16:0, 2ac | 23.23 | 173.94 | 124.015 | 2.63 | 1.08 |
| L. C18:1, 2ac | 24.54 | 3400.93 | 3029.802 | 51.47 | 55.92 |
| L. C18:1, 2ac* | 24.93 | 254.86 | 242.558 | 3.86 | 4.48 |
| Others | | 850.21 | 533.845 | 12.87 | 11.06 |
| **Total** | | **6607.34** | **5417.75** | **100.00** | **100.00** |

Others group involve no identify sophorolipids. *Data belongs to SL isomers*. Abbreviations:* Ac, acidic; L, lactonic and ac, amount of acetylation.


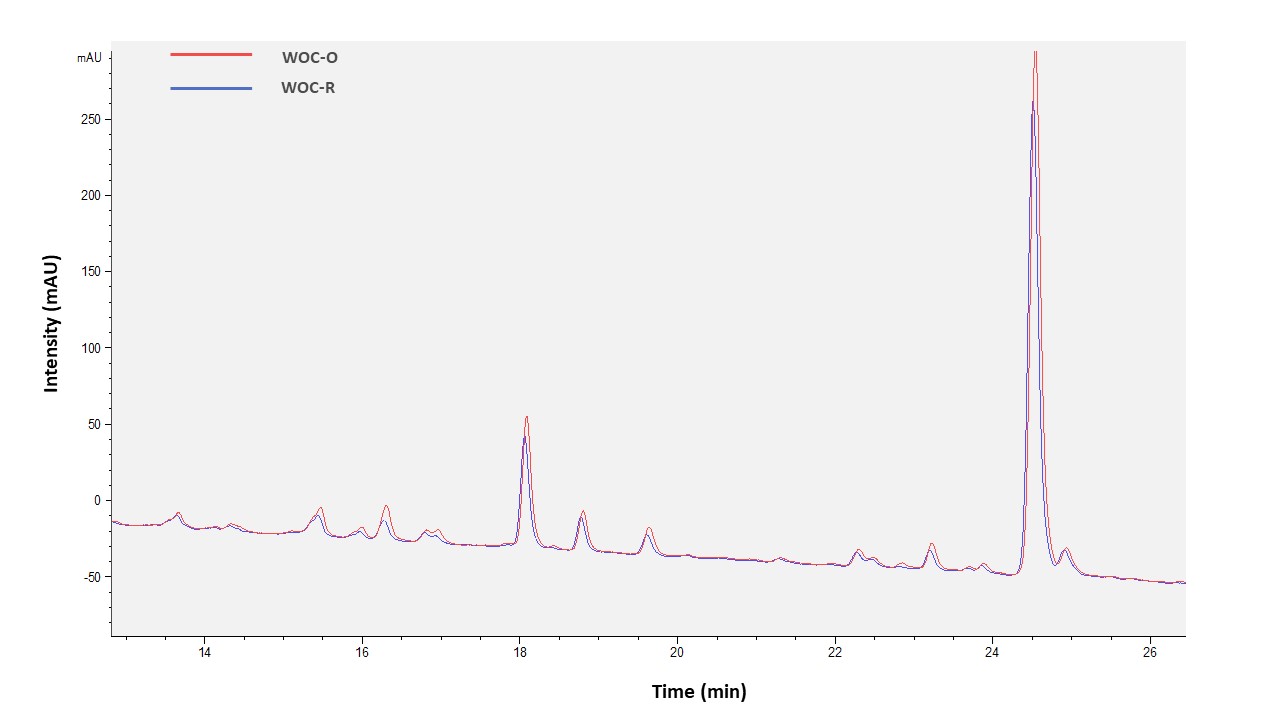


**Supplementary Figure S2.** Sophorolipids area comparison between WOC-O and WOC-R. HPLC-UV chromatogram.

**Supplementary Table S2.** ANOVA for the surface quadratic model when yeast growth was used as outcome.

|  |  | **Yeast growth**  (R^2^ = 63.33%) | |
| --- | --- | --- | --- |
| **Source** | **DF** | **Mean Square** | **p-value** |
| **Model** | 9 | 0.0598 | 0.0019* |
| $\boldsymbol{X}_{\mathbf{1}}$-Glucose | 1 | 0.0000 | 0.9741 |
| $\boldsymbol{X}_{\boldsymbol{2}}$-Nitrogen | 1 | 0.2261 | 0.0005* |
| $\boldsymbol{X}_{\boldsymbol{3}}$-Time | 1 | 0.2221 | 0.0005* |
| $\boldsymbol{X}_{\mathbf{1}}\boldsymbol{X}_{\boldsymbol{2}}$ | 1 | 0.0053 | 0.5368 |
| ${\boldsymbol{X}_{\mathbf{1}}\boldsymbol{X}}_{\mathbf{3}}$ | 1 | 0.0033 | 0.6276 |
| $\boldsymbol{X}_{\boldsymbol{2}}\boldsymbol{X}_{\mathbf{3}}$ | 1 | 0.0130 | 0.3377 |
| ${\boldsymbol{X}_{\boldsymbol{1}}}^{\boldsymbol{2}}$ | 1 | 0.0208 | 0.2278 |
| ${\boldsymbol{X}_{\boldsymbol{2}}}^{\boldsymbol{2}}$ | 1 | 0.0159 | 0.2896 |
| ${\boldsymbol{X}_{\boldsymbol{3}}}^{\boldsymbol{2}}$ | 1 | 0.0183 | 0.2568 |
| Residual | 23 | 0.0135 |  |
| Lack of Fit | 17 | 0.0174 | 0.0122* |

******Significant parameters (p < 0.05). Data was transformed to a basic 10 logarithm for calculations.*

**
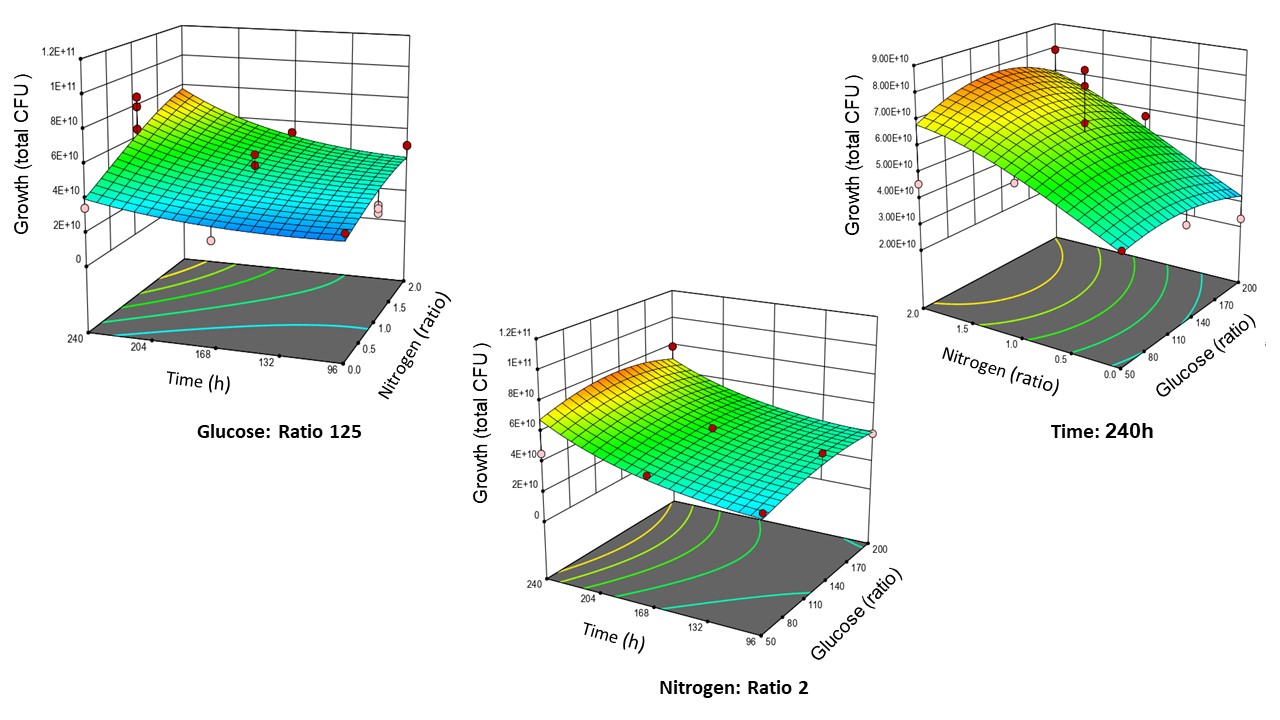
**

**a)**

**b)**

**c)**

**Supplementary Figure S3.** Response surface methodology for yeast growth. As influencing factors nitrogen and time with a p-value <0.05. (a) Influencing factors interaction at glucose central point (ratio 125); (b) Effect of time level +1 (240 h) on glucose and nitrogen parameters; (c) Nitrogen level +1 (ratio 2) influence on the evaluated parameters. Surface plots are colored from low (blue) to high (red).
